# NMR Investigation of the Copper(II)-Ciprofloxacin System

**DOI:** 10.1155/MBD.1999.1

**Published:** 1999

**Authors:** Iztok Turel, Janez Košmrlj, Bjørn Andersen, Einar Sletten

**Affiliations:** 1 University of Ljubljana Faculty of Chemistry and Chemical Technology Ašsskerčeva 5 Ljubljana 1000 Slovenia; 2 Department of Chemistry University of Bergen Allegt. 41 Bergen N-5007 Norway

## Abstract

A proton NMR study was performed on the copper(ll)-ciprofloxacin system. The proton relaxation times (T_1_) were determined from the titration data in acidic and basic media. In acidic medium the H5 signal is dramatically affected and it is assumed that copper is bonded to the quinolone through carbonyl and one of the carboxyl oxygens. Such bonding is in agreement with the X-ray literature data for the complex [Cu(cf)_1_]Cl_2_.6H_2_O isolated from the slightly acidic solution. There are additional significant changes in _1_T_1_ of H3′ and H5′ atoms which suggest that the terminal nitrogen atom of the piperazine ring system-N4′ also interacts with copper in the basic conditions. Thus it is plausible that more than one species are present in the solution at high pH values.


### NMR INVESTIGATION OF THE COPPER(II)-CIPROFLOXACIN SYSTEM

Iztok Turel*[1], Janez Košmrlj[1], Bjørn Andersen[2], and Einar Sletten[2]

[1] University of Ljubljana, Faculty of Chemistry and Chemical Technology, Aškerčeva 5, 1000 Ljubljana, Slovenia

[2] Department of Chemistry, University of Bergen, Allegt. 41, N-5007 Bergen, Norway

**Abstract**

A proton NMR study was performed on the copper(II)-ciprofloxacin system. The proton relaxation times ($T_1$) were determined from the titration data in acidic and basic media. In acidic medium the H5 signal is dramatically affected and it is assumed that copper is bonded to the quinolone through carbonyl and one of the carboxyl oxygens. Such bonding is in agreement with the X-ray literature data for the complex $[Cu(cf)_2]Cl_2{\cdot}6H_2O$ isolated from the slightly acidic solution. There are additional significant changes in $T_1$ of H3′ and H5′ atoms which suggest that the terminal nitrogen atom of the piperazine ring system-N4′ also interacts with copper in the basic conditions. Thus it is plausible that more than one species are present in the solution at high pH values.

### Introduction

A number of highly potent broad-spectrum quinolone antibacterial agents have been synthesized in the past few years, and many have been introduced into clinical practice. Ciprofloxacin (cf, Scheme 1a) is a typical member of this family (Chu and Fernandes, 1991; Koga et al., 1980) and nalidixic acid (nal, Scheme 1b) was the first compound from this group used as a drug. It is well known that the absorption of quinolones appears to be significantly reduced in the presence of various metal ions. The reason is probably the chelation between the quinolone and the metal ion (Cole et al., 1984; Polk, 1989; Kara et al., 1991).

Scheme 1: Chemical formulae of ciprofloxacin a) and nalidixic acid b).

a)

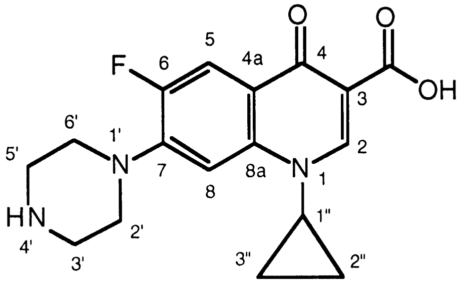

b)

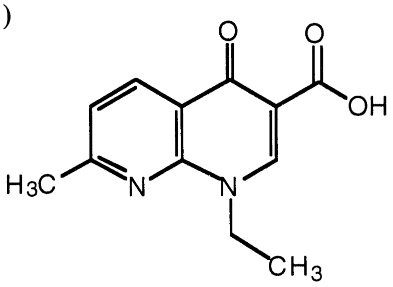

An NMR study of the proton relaxation times ($T_1$) was performed in the copper(II)-ciprofloxacin system. Our aim was to check for any differences in relaxation times between acidic and basic aqueous media which could suggest the presence of different bonding modes of the ligand. In a previous study of the Cu(II)-cf system we showed by potentiometric measurements that in the acidic region a 1:1 complex is formed, whereas a 1:2 complex prevailed at higher pH (Turel et al., 1996). Furthermore, it was unexpectedly revealed that the coordination of the second quinolone ligand in the 1:2 complex was somehow more favoured than that of the first one.

It has been established that copper(II) ions broaden the signals in the NMR spectra of those atoms found in the proximity of metal binding sites (Sterk et al., 1985; Eichhorn et al., 1966; Marzilli,1977; Kotowycz, 1974). $^{13}C$ NMR studies on the metal-quinolone systems have already been performed (Mendoza-Diaz and Panell, 1988; Wallis et al., 1996). The latter group proposed that the most important interaction of copper with ciprofloxacin in acidic solution is through the ring carbonyl and one of the carboxyl oxygens but in basic solution there is an additional interaction of the metal with the piperazine nitrogen atom. These conclusions have been drawn upon the observations of the broadening of the carbon resonances. It is well known that Cu(II) ions induce line broadening of $^{13}C$ resonances and that the results must be interpreted with great caution (Marzilli,1977; Kotowycz, 1974). Our $^{13}C$ results were comparable to those of Wallis, but we have decided to perform a detailed proton relaxation times analysis, which is expected to give more reliable results concerning the determination of binding sites for paramagnetic metal ions.

### Materials and Methods

*$^1H$ NMR Spectroscopy*. The $^1H$ NMR experiments were performed on a Bruker DRX 600 spectrometer operating at 600 MHz and on a Bruker DPX 300 instrument operating at 300 MHz. The spectra were assigned with the aid of different techniques (HMQC, DQFCOSY) and the results were in agreement with the literature data. All NMR experiments were recorded at 298 K. The solvent peak was suppressed by a 3-9-19 WATERGATE pulse sequence. One-dimensional spectra were recorded into 64K complex points with a 22 ppm spectral width, a 12 μs pulse width and a recycling delay of 2 s. The $T_1$ measurements were carried out using an inversion recovery sequence, in which the WATERGATE suppression sequence was incorporated. A list of 13 different delays in the range 0.01 to 10 s were used. The NMR data were processed on a PC (Pentium, 133 MHz) using the WIN-NMR version 960901.2 (Bruker). The spectra were multiplied by an exponential window function prior to Fourier transformation. Typically, 1 Hz was added to the line width. The 1D spectra were zero filled to 128 K. No baseline correction was needed. The proton spectra were referenced to the $H_2O$ resonance in water at 4.76 ppm at 298 K. An aqueous solution of cf·HCl (0.0778 M) was prepared (pH ca. 4). The basic solution was prepared by the addition of an NaOH solution to reach the final pH 11. To both solutions successive aliquots of *ca*. 77.0 mM $CuCl_2$ were added with a micropipette.

### Results and Discussion

The effect on the proton spin-lattice relaxation rates ($1/T_1$) *vs*. Cu(II) concentration (acidic solution) is plotted in Fig. 1a. The H5 signal is dramatically affected compared to the other ring proton signals due to a dipolar interaction between the unpaired electron on Cu(II) and the H5 nucleus. This result is in agreement with the crystal structure of the $[Cu(cf)_2]Cl_2 \cdot 6H_2O$ complex (Turel et al., 1994) obtained from acidic medium where bonding through carbonyl and one of the carboxyl oxygens was found. The second possible coordination geometry in this system could be the chelate bonding of the metal to the carboxylic group. The dipolar interaction is proportional to $r^{-6}$ where r is the distance between the paramagnetic center and the proton. The relative effect on the relaxation rates between the two sites calculated on the basis of this model is 11:1, which is of the right order of magnitude as compared to the experimental rates.

The linear relationship of relaxation rate *vs*. metal ion concentration indicates the presence of only one major species in acidic solution.

Titrations were also performed in basic medium and the results are shown in Fig. 1b. The quinolone hydrogen atoms (H5, H8 and H2) are affected similarly as in the acidic medium indicating the same type of coordination. In addition there is a significant change in $T_1$ of H3′and H5′ atoms compared to H2 and H8 which suggests that N4′ also interacts with copper under these conditions.

The non-linear relationship of relaxation rate *vs*. metal ion concentration indicates that more than one species are present at high pH. It is interesting to note that in the crystal structure of the silver-quinolone (pefloxacin) complex (prepared from basic medium) (Baenzinger et al., 1986) both carboxylic oxygens and the piperazine nitrogen are all involved in bonding to the metal. In this structure two silver ions are joined in dimeric pairs by bridging coordinating carboxyl groups of two pefloxacin molecules.

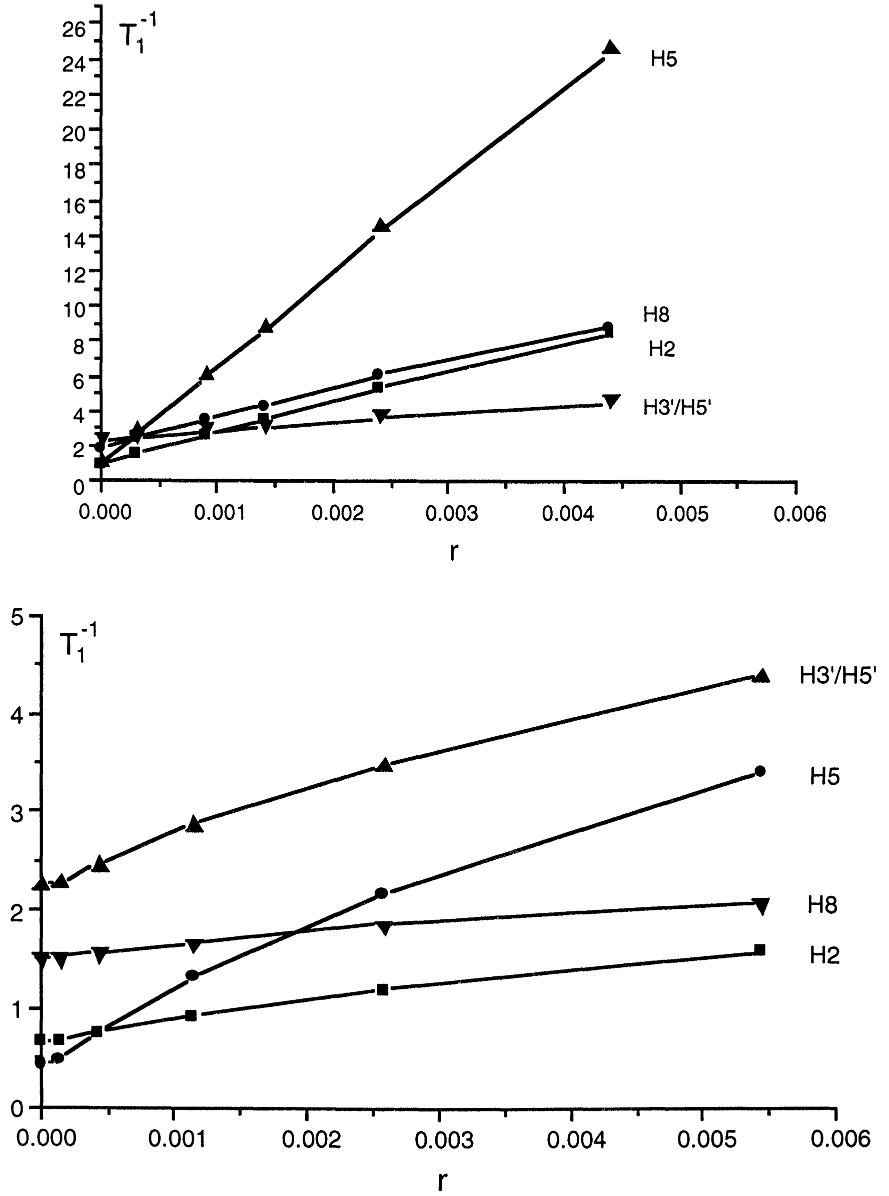

Figure 1. Effect of Cu(II) concentration on spin-lattice relaxation rates ($1/T_1$) [$s^{-1}$] in (a) acidic and (b) basic solutions of cf (r = n(Cu(II) / n(cf)), c(cf)=0.0778 M).

Copper(II) coordination to nalidixic acid (Scheme 1b) has been studied by $^{13}C$ line-broadening measurements (Mendoza-Diaz and Panell, 1988). The authors propose that the $Cu(aq)^{2+}$ ion forms a chelate with the carboxylic group in the absence of additional ligands, since the carbonyl carbon is virtually unaffected. This result differs from that obtained in the present study. While the two systems are not equivalent, it is not immediately apparent why this difference should exist.

**Acknowledgements**
The Ministry of Sciences and Technology, Republic of Slovenia is thanked for financial support (grant No. Z1-8601-0103-96). B.A. gratefully acknowledges a fellowship from the Norwegian Research Council.

**References**

1. Baenzinger, N.C., Fox, C.L., Modak, S.L. *Acta Cryst.*, 1986; **C42**: 1505.
2. Chu, D.T.V., Fernandes, P.B. *Adv. Drug Res.*, 1991; **21**: 42.
3. Cole, A., Goodfield, J., Williams, D.R. and Midgley, J.M. *Inorg. Chim. Acta*, 1984; **92**: 91, and the references therein.
4. Eichhorn, G.L., Clark, P. and Becker, E. D. *Biochem.*, 1966; **5**: 245.
5. Kara, M., Hasinoff, B.B., McKay, D.W. and Campbell, N.R.C. *Br. J. Clin. Pharmac.*, 1991; **31**: 257, and the references therein.
6. Koga, H., Itoh, A., Murayama, S., Suzue, S. and Irikura, T. *J. Med. Chem.*, 1980; **23**: 1358.
7. Kotowycz, G. *Can. J. Chem.*, 197;4**52**: 924.
8. Marzilli, L.G. in *Prog. Inorg. Chem.*, (ed. S. J. Lippard), 1977; **Vol.23**: 255, John Wiley & Sons, New York, and the references therein.
9. Mendoza-Diaz, G. and Panell, K. H. *Inorg. Chim. Acta*, 1988; **152**: 77.
10. R. E. Polk, *Am. J. Med.*, 1989; **87**: 5A.
11. Sterk, H., Braun, M., Schmut, O., Feichtinger, H. *Carbohydrate Research*, 1985; **145**: 1.
12. Turel, I., Leban, I., Bukovec, N., *J. Inorg. Biochem.*, 1994; **56**: 273.
13. Turel, I., Bukovec N. and Farkas, E. *Polyhedron*, 1996;**15**: 269.
14. Wallis, S.C., Gahan, L.R., Charles, B.G., Hambley, T.W. and Duckworth, P.A. *J. Inorg. Biochem.*, 1996; **62**:1.

**Received: November 4, 1998 - Accepted: December 3, 1998 - Received in revised camera-ready format: December 4, 1998**

